# Moderate-Load Muscular Endurance Strength Training Did Not Improve Peak Power or Functional Capacity in Older Men and Women

**DOI:** 10.3389/fphys.2017.00743

**Published:** 2017-09-26

**Authors:** Simon Walker, Guy G. Haff, Keijo Häkkinen, Robert U. Newton

**Affiliations:** ^1^Faculty of Sport and Health Sciences and Neuromuscular Research Center, University of Jyväskylä, Jyväskylä, Finland; ^2^Centre for Exercise and Sports Science Research, Edith Cowan University, Joondalup, WA, Australia

**Keywords:** aging, resistance, hypertrophy, intensity, walking, stair climb, timed-up-and-go, rest interval

## Abstract

The present study determined the effects of muscular endurance strength training on maximum strength and power, functional capacity, muscle activation and hypertrophy in older men and women. Eighty-one men and women acted as an intervention group while 22 acted as non-training controls (age range 64–75 y). Intervention training included super-sets (i.e., paired exercises, immediately performing the second exercises following completion of the first) with short rest intervals (30–60 s between sets) at an intensity of 50–60% one-repetition maximum (1-RM) for 15–20 repetitions. Concentric leg press actions measured maximum strength (1-RM) and concentric peak power. Functional capacity was assessed by maximum speed walking tests (i.e., forward walk, backward walk, timed-up-and-go, and stair climb tests). Quadriceps muscle activation was assessed by surface electromyogram and twitch interpolation technique. Vastus lateralis cross-sectional area was measured by panoramic ultrasound. Compared to control, the intervention groups increased maximum strength (1-RM; men: 10 ± 7% vs. 2 ± 3%, women: 14 ± 9% vs. 1 ± 6% both *P* < 0.01) and vastus lateralis cross-sectional area (men: 6 ± 7% vs. −3 ± 6%, women: 10 ± 10% vs. 0 ± 4% both *P* < 0.05). But there were no between-group differences in peak power, muscle activation or functional capacity (e.g., stair climb; men: −5 ± 7% vs. −4 ± 3%, women: −5 ± 6% vs. −2 ± 5% both *P* > 0.05). While benefits occurred during muscular endurance strength training, specific stimuli are probably needed to target all aspects of age-related health.

## Introduction

Combating the age-associated loss of strength, power and muscle mass through strength training is of great importance to maintain functional capacity (Raj et al., 2010) and prevent certain diseases (e.g., Srikanthan and Karlamangla, 2011). There is an abundance of literature supporting the use of progressive high-load strength training to increase strength and muscle mass (Fiatarone et al., 1990; Häkkinen et al., 1998; Harridge et al., 1999; Walker et al., 2014). In addition to strength and muscle mass, important aspects of function and health that may be compromised during aging, such as power and rate of force development that are important factors in functional capacity (Bassey et al., 1992; Steib et al., 2010), can be enhanced through strength training in older individuals (Henwood and Taaffe, 2005; Lovell et al., 2010).

There is emerging evidence that low-load strength training may be similarly effective to high-load training in previously untrained older adults for gains in strength and muscle mass (Taaffe et al., 1996; Tanimoto and Ishii, 2006; Van Roie et al., 2013), which is reflected by results of a recent meta-analysis (Csapo and Alegre, 2016). Furthermore, results of another meta-analysis suggest that improvements in systolic blood pressure and other markers of metabolic syndrome were more likely to be favorable when the training program included a higher total number of repetitions and was of longer duration (>10 weeks) (Strasser et al., 2010). These findings may indicate that a better overall impact (i.e., broader range of adaptations) may occur during strength training that is defined as muscular endurance strength training; i.e., moderate-loads (40–60% of maximum), ~10–15 repetitions per sets and is particularly characterized by very short inter-set rest periods (~30–60 s; Clayton et al., 2015).

Certainly the prospect of combating several facets of impaired age-related functional capacity and metabolic health within one training regime is an attractive notion. Indeed, Campos et al. (2002) demonstrated that a “high rep” group performing 20–28 repetitions for two sets (1 min rest) improved maximum strength, muscular endurance and aerobic capacity, giving a broader range of improvements compared to the “low and intermediate rep” groups who performed 3–5 repetitions for four sets (3 min rest) and 9–11 repetitions for three sets (2 min rest), respectively. However, Walker et al. (2015) demonstrated that performing contractions in a controlled manner (i.e., 2 s concentric and 2 s eccentric) during training did not lead to improvements in leg press maximum concentric peak power in older individuals. Given that lower-limb extension power is an important determinant of functional capacity (Bassey et al., 1992), it may be that moderate-load muscular endurance strength training is not best suited to improve power and perhaps functional capacity in turn.

To the authors' knowledge no study has investigated the effects of moderate-load muscular endurance strength training on neuromuscular performance and functional capacity in older individuals. Consequently, before any recommendations regarding training program design can be provided effects on the aforementioned performance and neuromuscular outcome measures must be determined. Therefore, the aim of the present study was to determine the effects of a 12-week moderate-load muscular endurance strength training period on maximum strength and power, muscle activation and muscle mass and functional capacity in both older men and women.

## Materials and methods

### Trial design

This was a randomized, four-group parallel study. The study was conducted according to the Declaration of Helsinki and was approved by the ethical committee of the University of Jyväskylä, Finland. Participants were measured before and after a 12-week intervention or control period.

### Participants

Participants were healthy 65–75-year-old men and women. Exclusion criteria were; (1) regular aerobic exercise (>180 min week), (2) any previous strength training experience, (3) Body Mass Index >37, (4) serious cardiovascular disease or lower-limb injuries/disease that may lead to complications during exercise or affect the ability to perform testing and training, (5) use of walking aids, (6) use of medication that affect neuromuscular or endocrine systems, (7) previous testosterone-altering treatment, and (8) smoking. Although classed as “healthy,” most participants were taking some form of medication for various conditions, in particular high blood pressure and/or high cholesterol in several cases. Typical medications that were taken included; blood pressure medication (experimental *n* = 28 and control *n* = 7), cholesterol medication (experimental *n* = 15 and control *n* = 4), blood glucose medication (experimental *n* = 6 and control *n* = 0), thyroid medication (experimental *n* = 11 and control *n* = 0) and beta-blockers (experimental *n* = and control *n* = 2).

### Participant flow and baseline data

**Figure 1:**
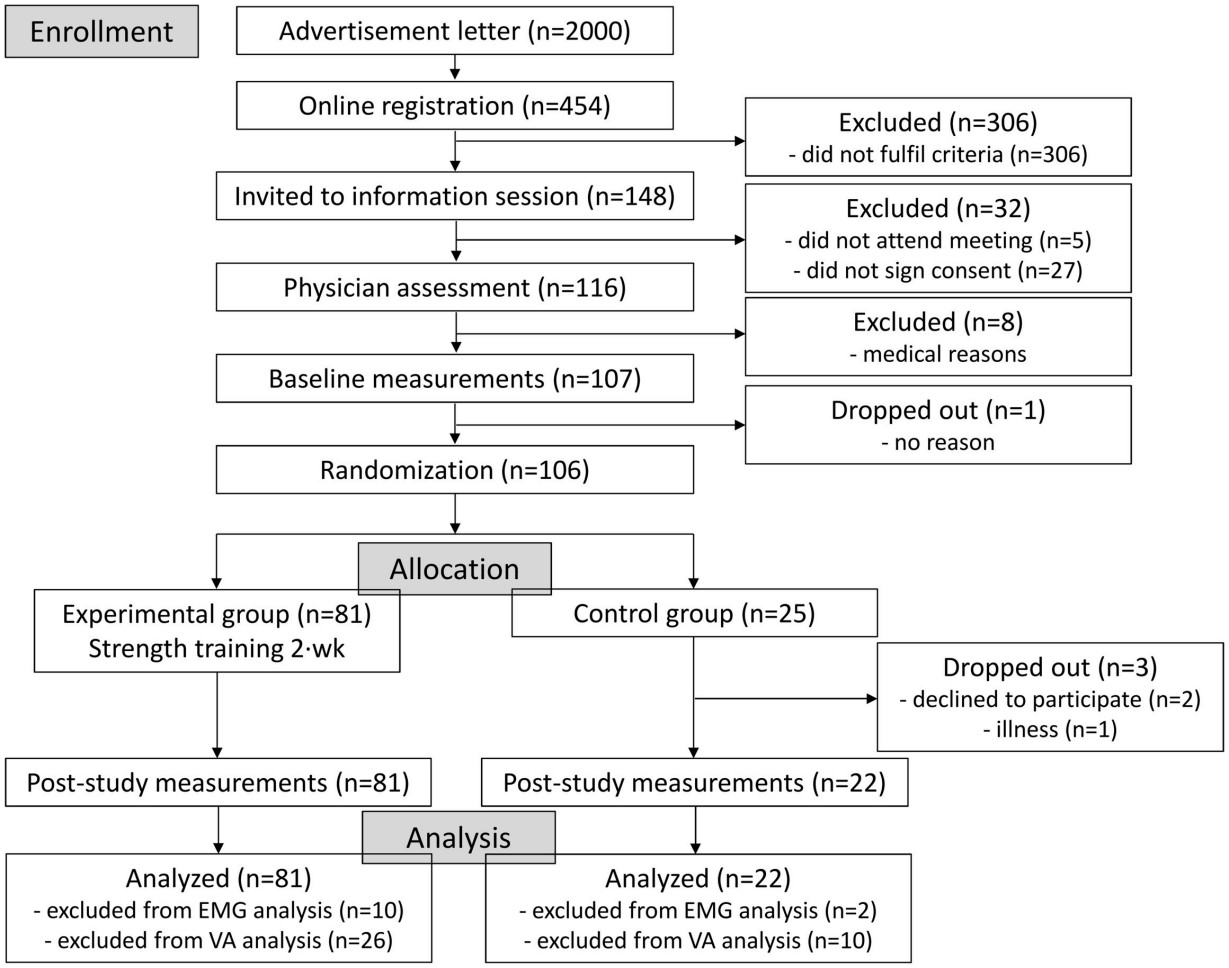
Flow chart of study enrolment, allocation and analysis. EMG, electromyography; VA, voluntary activation level.

The recruitment process and exclusion of participants is shown in Figure 1. Initially, advertisement letters were posted to 2,000 individuals in the Jyväskylä region whose contact details were randomly selected by the Population Register Centre, Finland. Potential participants registered to the study by completing an online researcher-designed questionnaire (*n* = 454) containing information regarding leisure-time physical activity levels (min·week), general health/medical history and current medications. After assessing the eligibility of the registered individuals for lower-limb injuries and physical activity levels, potential participants were invited to an information session (*n* = 148). Each participant was carefully informed of the study design and potential risks before the study, after which they provided written consent (*n* = 116). Prior to measurements, the participants were examined by a physician including a resting electrocardiogram and were cleared to perform rigorous exercise (*n* = 108).

As this arm of the study is part of a larger randomized controlled trial (NCT02413112), with 3 intervention groups performing different interventions after the completion of this initial 12-week period, the sample size of the intervention and control groups are not equivalent here. After testing, data was checked and due to technical failure several participants' electromyography and voluntary activation level data were excluded from the analysis (as noted in Figure 1). Baseline characteristics of the participants in each group are shown in Table 1, with the only differences observed between men and women in height and body mass.

**Table 1:** Baseline characteristics and leisure-time physical activity of the subjects (mean ± SD).

	**Experimental groups**		**Control groups**	
	**Men (*n* = 35)**	**Women (*n* = 46)**	**Men (*n* = 12)**	**Women (*n* = 10)**
Age (years)	69.8 ± 2.4	68.6 ± 2.0	69.9 ± 2.8	69.0 ± 2.7
Height (m)	1.76 ± 0.04	1.61 ± 0.05^*^	1.74 ± 0.04	1.59 ± 0.04^*^
Body mass (kg)	88.7 ± 13.4	72.7 ± 10.9^*^	81.3 ± 7.3	65.4 ± 8.1^*^
Body mass index (kg·m^2^)	28.8 ± 4.0	27.9 ± 4.0	26.8 ± 2.3	25.7 ± 2.7
Physical activity (min·week)	95 ± 63	105 ± 59	101 ± 56	115 ± 70

* *Difference compared to men, P < 0.05*.

### Adverse events

There were no adverse effects reported during the training and testing processes; one control subject had a recurrence of a previous illness during the study.

### Randomization

After baseline testing, the remaining participants (*n* = 106) were allocated an identification number and a computer-generated random number sequencer was used to allocate each participant into one of four groups. Block randomization was performed for the first 100 participants selected, ensuring that each group contained 25 participants. Thereafter, the remaining non-allocated 6 participants were randomly allocated to the intervention groups. For the purposes of the present study, the three intervention groups were combined since they all followed the same initial intervention.

### Familiarization session

Approximately 7 days before testing the participants visited the laboratory to become familiarized with all test protocols. Here, all test devices were set according to individual participant's anthropometry and practice trials were performed. Electrode locations for electromyography (EMG) recordings were marked by indelible ink tattoo to allow accurate replacement during all test sessions. Participants were required to perform these tests with perfect technique by the cessation of the session, with 5–10 warm-up contractions performed prior to maximal effort trials. The participants were also allowed 2–3 practice trials of each functional capacity test at the end of the session. The same instructions and performance requirements were followed in the familiarization session as the following performance tests.

### Intervention

The intervention group performed muscular endurance strength training twice-per-week for 12 weeks with at least 48 h between sessions and each session was supervised by experienced gym instructors. All exercises were performed on commercially available weight-stack strength equipment (Precor Vitality Series™, Precor Inc., UK). The 12-week program was divided into a 4-week initiation phase and an 8-week super-set training phase (Table 2). The primary goal of the initiation phase was to teach the participants correct technique for all exercises and to progressively increase the loads used so that a true 16-RM load would be determined in week 4. The primary goal of the super-set phase was to limit rest periods to challenge the anaerobic and aerobic energy systems to maintain work output. Intensity for all upper and lower-limb exercises was ~50–60% 1-RM. This type of training program is in-line with those classified as muscular endurance strength training (Clayton et al., 2015), and consists of lower load, higher number of repetitions per set and reduced inter-set/exercise rest periods in comparison to typical high-intensity strength training interventions in the literature. All subjects were required to perform all repetitions using a tempo of 2 s concentric and 2 s eccentric phase and the selected load was aimed to induce volitional concentric failure in the final repetition of the final set. Therefore, the precise load was adjusted between-sets if the supervisor observed that it was too light or too heavy to perform the exercise in such a way as to fulfill these aims. All participants were required to complete at least 21 out of 24 training sessions prior to testing. Participants in the non-training control group were instructed to maintain their normal physical activity throughout the study period. All participants recorded their daily leisure-time physical activity levels in diaries.

**Table 2:** Twelve-week experimental strength training protocol.

**Weeks**	**Session 1**			**Session 2**		
	**Exercise**	**Set × reps**	**Rest periods**	**Exercise**	**Set × reps**	**Rest periods**
1–4	Leg press	2 × 16–20	60 s between sets, 120–180 s between exercises	Leg press	2 × 16–20	60 s between sets, 120–180 s between exercises
	Knee extension			Knee extension		
	Knee flexion			Knee flexion		
	Chest press			Shoulder press		
	Lat pulldown			Seated row		
	Tricep pushdown			Bicep curl		
	Ab curl			Seated calf-raise		
	Back extension			Ab curl		
				Back extension		
5–12	Leg press + chest press	3 × 14–16	0 s between super-sets, 30 s between sets, 120–180 s between exercises	Leg press + seated row	3 × 14–16	0 s between super-sets, 30 s between sets, 120–240 s between exercises
	Knee extension + lat pulldown	3 × 14–16		Knee extension + shoulder press	2 × 14–16	
	Knee flexion + tricep pushdown	2 × 14–16		Knee flexion + bicep curl	3 × 14–16	
	Ab curl + back extension	2 × 14–16		Seated calf-raise + shoulder raise	2 × 14–16	
				Ab curl + back extension	2 × 14–16	

*Reps, repetitions; Lat, Latissimus dorsi; Ab, abdominal. Session 1 refers to the first session performed each week, while Session 2 refers to the following session of the same week. These sessions were separated by at least 48 h rest*.

### Primary outcome measures

#### Dynamic leg press strength

Concentric bilateral leg press one-repetition maximum (1-RM) and maximum concentric peak power was measured ~7 d before and after the 12-week training period. Briefly, following warm-up, single repetitions with increments of 5 kg were performed until the participants could no longer fully extend their hips and legs (full extension = 180°). Each trial was separated by 1.5 min. Thereafter, half of the identified 1-RM load (50% 1-RM) was removed and participants performed three leg press trials separated by 1 min of rest. Here, the participants were instructed to extend the hips and knees “as fast as possible” and peak power was calculated over a period of 50 ms using customized scripts and the following equation;

$$\begin{array}{l} concentric\;peak\;power\;=\;\left(load\times 9.81\right)\times \left(displacement/time\right) \end{array}$$

All data was relayed to a pc via an AD converter (Micro 1401, Cambridge Electronic Design, UK) and recorded using Signal 4.04 software (Cambridge Electronic Design, UK). Data was sampled at 2,000 Hz and filtered by a 10-Hz low-pass filter (fourth-order Butterworth) and the best trials were used in further analyses.

#### Functional capacity

Four maximal walking tests performed were included in the assessment of functional capacity; (1) 7.5 m forward walk, (2) 7.5 m backward walk, (3) Timed Up-and-Go test (TUG), and (4) loaded 10-stair climb test. Participants were instructed to perform the tests “as fast as possible without compromising safety.” Each test was recorded by photocells except TUG, which used a contact mat positioned under the chair to determine rise from and return to the chair. The best performance from two acceptable trials was used in the analyses and the sum result from both directions was used for TUG. The participants were not allowed to use their arms to assist in the chair rise or return. During the 10-stair climb test, the participants carried one bag of 5 kg (women) or 10 kg (men) and were instructed to maintain an extended elbow position and prevent arm-swinging during the ascent.

### Secondary outcome measures

#### Isometric knee extension and plantarflexion strength

Unilateral isometric knee extension force of the right leg was measured using a custom-built isometric force chair. Inelastic straps were used to secure the participant with both hip and knee angles of 110°. Participants were instructed to kick “as fast and as hard as possible” and maintain their maximum force for ~3 s. The force signal was sampled as described in the leg press trials with the highest force used in further analysis. Three trials were performed with a fourth trial performed if improvements were more than 5%. Thereafter, two additional maximum isometric knee extension trials were performed with femoral nerve stimulation delivered during the force plateau and 2 s after contraction cessation (see section Voluntary Activation Level). Maximum force was measured and then converted to torque by taking into account the lever arm distance from the knee joint-center to the ankle strap (KE_MVC_).

Bilateral isometric plantarflexion force was assessed in a seated position by a custom-built plantarflexion device with knees flexed to ~90° using similar methods to Unhjem et al. (2015). The balls of the feet were positioned on a shelf connected to the strain gauge (90° ankle joint-angle) and the knees were held in-place by a cushioned board. Participants performed 3–4 isometric plantarflexion actions following the same instructions as for the knee extension trials. Maximum force was measured and then converted to torque by taking into account the lever arm distance from the ankle joint-center to the shelf connected to the strain gauge (PF_MVC_). Torque was also normalized to the cross-sectional area of the measured muscles that influence these actions (KE_norm_ and PF_norm_).

#### Maximum voluntary muscle activity

Bipolar Ag/AgCl electrodes (5 mm diameter, 20 mm inter-electrode distance, common mode rejection ratio >100 dB, input impedance >100 MΩ, baseline noise <1 μV rms) were positioned following shaving and skin abrasion on the vastus lateralis (VL) and medialis (VM) of the right leg according to SENIAM guidelines. Raw EMG signals were sampled at 2,000 Hz and amplified at a gain of 500 (sampling bandwidth 10–500 Hz). Raw signals were sent from a hip-mounted pack to a receiving box (Telemyo 2400R, Noraxon, Scottsdale, USA), then were relayed to an AD converter (Micro1401, Cambridge Electronic Design, UK) and recorded by Signal 4.04 software (Cambridge Electronic Design, UK). Offline, EMG signals were band-pass filtered at 20–350 Hz and root mean square was obtained from approx. 65° to full leg extension (i.e., 180°) during dynamic leg press (1-RM and maximum power) actions. Values are taken from the best trials in each performance measure.

#### Voluntary activation level

Rectangular pulses (400 V) of 200 μs were delivered by a constant current stimulator (Model DS7AH, Digitimer Ltd., UK) to the femoral nerve of the right leg through 5 cm^2^ self-adhesive electrodes (Polar Trode, Niva Medical Ltd., Espoo, Finland) placed in the femoral triangle either side of the nerve, which was identified by palpating and identifying the femoral artery. Current intensity was gradually increased until no further increases were observed in peak-to-peak M-wave amplitude of VL and VM. To ensure maximal activation, an additional 20% current was used during subsequent stimulations. Single twitches were delivered in a resting condition to determine peak-to-peak maximum M-wave amplitude and duration. Single twitches were also delivered about the maximum torque during isometric knee extension trials and 2 s after contraction cessation to determine voluntary activation level according to Merton's (1954) interpolated twitch technique, as previously described (Walker et al., 2014).

#### Muscle cross-sectional area

Muscle cross-sectional area (CSA) measurements of the right leg were taken 1–2 days prior to dynamic leg press performance tests and 6–7 days after the final training session to account for any exercise-induced swelling. CSA of the vastus lateralis, vastus intermedius, gastrocnemius medialis and lateralis was assessed by B-mode axial-plane ultrasound (model SSD-α10, Aloka Co Ltd., Tokyo, Japan) using a 10 MHz linear-array probe (60 mm width) coated with water-soluble transmission gel with the extended-field-of-view mode (23 Hz sampling frequency). Indelible ink tattoos on the medial and lateral sides of the target muscles ensures accurate replacement of scanning track. Oriented in the axial-plane, the probe was moved manually with a slow and continuous movement from medial to lateral along a marked line on the skin. Great care was taken to diminish compression of the muscle tissue. Images were obtained throughout the movement. As the orientation of each image relative to adjacent images is known, the software builds a composite image. Four panoramic CSA images were taken at; (1) 50% femur length from the lateral aspect of the distal diaphysis to the greater trochanter and (2) 30% lower-limb length from the lateral articular cleft between the femur and tibia condyle to the lateral malleolus following methods used by Walker et al. (2016) for the quadriceps and Rosenberg et al. (2014) for the gastrocnemii. Upon visual inspection of the composite images three were selected to undergo further analysis. CSA was determined by manually tracing along the border of each muscle using Image-J software (version 1.37, National Institute of Health, USA). The mean of the two closest values for each muscle were taken as the CSA result.

#### Total body composition

Participants fasted overnight for 12 h and were instructed to drink 0.5 l of water 1 h before measurements. After determination of height by a fixed wall-mounted scale, participants underwent full body scanning by dual-energy X-ray absorptiometry (DXA) in minimal clothing (LUNAR Prodigy Advance with encore software version 9.3, GE medical systems, USA). The legs were separated by a polystyrene block and secured by inelastic straps about the ankles. Total body fat mass and fat-free mass, as well as fat-free mass of the legs was determined using software-generated analysis.

### Sample size

The sample size was estimated from the reported effect sizes in Liu and Latham's (2009) meta-analysis for maximum strength and functional capacity. To ensure an 80% probability that a treatment difference could be detected for a 5% level of significance, a sample size of 18 and 22 in each group was necessary to detect changes in strength and functional capacity, respectively.

### Statistical methods

All data are presented as means and standard deviations (±SD). All statistical methods were performed using IBM SPSS statistics 24 software. The Kolmogorov-Smirnov test was used to test normality and Levene's test was used to analyze homogeneity of variance. One-way analysis of variance with Bonferroni *post-hoc* tests was used to assess differences between the sex groups at baseline. Thereafter, statistical analyses were performed separately for men and women. All outcome measures were assessed using analysis of covariance (ANCOVA; 2 time × 2 group) with baseline values and the change in leisure-time physical activity used as covariates and Bonferroni *post-hoc* tests. Between-group ANCOVA-derived (adjusted) mean differences, *p*-values and 95% confidence intervals (CI) are reported. Also, to determine whether within-group changes were significant, paired *t*-tests were used on absolute values with delta% values reported. Effect sizes (Hedges' g) were calculated for the unadjusted mean differences between the intervention and control groups, where small (<0.3), medium (0.3–0.8), and large (>0.8) effect sizes were identified. Statistical significance was accepted when *P* < 0.05.

Performance for the whole group (i.e., intervention and control) during the familiarization session was; 1-RM: ~97%, peak power: ~100%, KE_MVC_ ~99%, PF_MVC_ ~107% of the baseline values. Reliability for the performance measures between the familiarization session and baseline measures were; 1-RM 0.97 and 5.5%, peak power 0.94 and 11.2%, KE_MVC_ 0.89 and 9.6%, PF_MVC_ 0.87 and 9.7%, forward walk 0.82 and 6.3%, backward walk 0.81 and 8.3%, TUG 0.89 and 3.2%, 10-stair climb 0.96 and 3.2%, and CSA 0.94 and 4.2% for Intra-class correlation coefficient (r) and coefficient of variation (%), respectively.

## Results

### Intervention adherence

Adherence to the intervention was 97 ± 4%; with nine subjects completing 21 of the allocated training sessions, ten subjects completing 22, twenty-one subjects completing 23 and forty-one subjects completing all 24 sessions.

### Strength and muscle activity

In men (Table 3), a significant main effect for group was observed for leg press 1-RM (*F* = 11.4, *P* = 0.002, 95% CI = 1.9–7.5) with training-induced improvements of 10 ± 7% (*P* < 0.001). A significant main effect for group was also observed in maximum isometric knee extension torque (*F* = 4.7, *P* = 0.036, 95% CI = 0.5–15.0) with improvements of 7 ± 9% (*P* < 0.001). In women (Table 4), a significant main effect for group was observed for leg press 1-RM (*F* = 9.0, *P* = 0.004, 95% CI = 1.7–8.7) with significant training-induced improvements of 14 ± 9% (*P* < 0.001).

**Table 3:** Outcome measures before and after the study and between-group comparisons in the men (mean ± SD).

	**Before**		**After**		Δ		**ANCOVA**		
	**Exp**.	**Con**.	**Exp**.	**Con**.	**Exp**.	**Con**.	**Adj. mean diff**.	***P*-value**	**95% CI**
**STRENGTH**									
Leg press 1-RM (kg)	**141 ± 27**	**139 ± 17**	**153 ± 24**	**143 ± 16**	**12 ± 8**	**3 ± 4**	**4.72**	**0.002**	**1.89 to 7.54**
Leg press peak power (W)	1,633 ± 373	1,612 ± 310	1,621 ± 336	1,719 ± 252	−12 ± 270	107 ± 180	−36.4	0.418	−126 to 53
KE_MVC_ (Nm)	**198 ± 32**	**192 ± 37**	**212 ± 31**	**193 ± 40**	**14 ± 18**	**1.7 ± 13**	**7.77**	**0.036**	**0.5 to 15.0**
KE_norm_ (Nm·cm^2^)	5.69 ± 1.25	5.58 ± 1.24	5.85 ± 1.24	5.81 ± 1.38	0.2 ± 0.8	0.2 ± 0.3	−0.18	0.901	−0.31 to 0.27
PF_MVC_ (Nm)	378 ± 66	343 ± 85	393 ± 83	375 ± 77	15 ± 39	32 ± 44	−5.1	0.528	−21.3 to 11.1
PF_norm_ (Nm·cm^2^)	20.7 ± 3.1	20.1 ± 5.8	21.1 ± 3.9	21.6 ± 5.0	0.5 ± 2.4	1.5 ± 2.8	−0.61	0.219	−1.6 to 1.6
**MUSCLE ACTIVITY**									
1-RM VL (μV)	196 ± 84	181 ± 60	240 ± 96	202 ± 58	44 ± 44	21 ± 32	3.17	0.708	−13.9 to 20.3
1-RM VM (μV)	198 ± 76	233 ± 81	244 ± 110	246 ± 81	47 ± 57	12 ± 56	0.56	0.957	−20.7 to 20.6
Power VL (μV)	213 ± 106	193 ± 79	245 ± 110	207 ± 71	33 ± 51	14 ± 62	11.20	0.316	−11.2 to 33.6
Power VM (μV)	189 ± 72	236 ± 84	232 ± 109	269 ± 124	42 ± 74	33 ± 78	4.72	0.769	−27.6 to 37.1
Voluntary activation (%)	93.3 ± 3.7	95.2 ± 3.0	93.5 ± 3.6	95.9 ± 2.6	0.4 ± 3.2	0.8 ± 2.4	−1.17	0.152	−2.81 to 0.47
M-wave duration (ms)	9.9 ± 2.9	11.4 ± 2.1	9.9 ± 2.9	11.0 ± 2.1	−0.03 ± 2.6	−0.47 ± 0.9	0.37	0.519	−0.78 to 1.52
**BODY COMPOSITION**									
Total FFM (kg)	62.0 ± 7.1	60.0 ± 3.4	62.8 ± 6.8	60.3 ± 3.7	0.8 ± 1.5	0.2 ± 0.7	0.33	0.240	−0.3 to 0.9
Leg FFM (kg)	**19.4 ± 2.3**	**19.3 ± 0.9**	**19.8 ± 2.3**	**19.2 ± 1.6**	**0.4 ± 0.9**	**−0.1 ± 1.0**	**0.38**	**0.042**	**0.02 to 0.7**
Fat mass (kg)	26.2 ± 8.2	21.7 ± 5.7	25.4 ± 8.1	21.9 ± 5.6	−0.9 ± 1.6	0.2 ± 1.6	−0.10	0.765	−0.8 to 0.6
VL CSA (cm^2^)	**16.7 ± 2.6**	**14.7 ± 1.8**	**17.7 ± 3.1**	**14.3 ± 2.0**	**0.9 ± 1.2**	**−0.5 ± 0.9**	**0.64**	**0.009**	**0.17 to 1.1**
VI CSA (cm^2^)	19.7 ± 5.2	19.2 ± 3.0	20.3 ± 5.0	18.6 ± 2.5	0.6 ± 2.7	−0.6 ± 1.2	0.76	0.118	−0.2 to 1.72
GM CSA (cm^2^)	12.5 ± 2.6	11.9 ± 2.5	12.7 ± 3.0	11.8 ± 2.8	0.2 ± 0.9	−0.1 ± 0.8	0.18	0.293	−0.16 to 0.52
GL CSA (cm^2^)	6.4 ± 1.7	5.6 ± 1.8	6.4 ± 1.6	5.8 ± 1.8	0.09 ± 1.1	0.2 ± 0.4	0.11	0.560	−0.27 to 0.49
**FUNCTIONAL CAPACITY**									
Forward walk (s)	2.72 ± 0.41	2.55 ± 0.42	2.57 ± 0.35	2.43 ± 0.25	−0.15 ± 0.23	−0.17 ± 0.24	0.03	0.495	−0.06 to 0.12
Backward walk (s)	3.52 ± 0.72	3.24 ± 0.52	3.21 ± 0.49	3.06 ± 0.31	−0.31 ± 0.40	−0.22 ± 0.31	0.04	0.583	−0.12 to 0.21
Timed-up-and-go (s)	8.68 ± 1.04	8.36 ± 0.84	8.14 ± 0.83	8.05 ± 0.67	−0.54 ± 0.47	−0.47 ± 0.37	−0.03	0.696	−0.19 to 0.13
Stair climb (s)	3.08 ± 0.37	3.00 ± 0.40	2.91 ± 0.29	2.93 ± 0.36	−0.17 ± 0.22	−0.11 ± 0.11	0.00	0.996	−0.08 to 0.08
Leisure-time PA (min·week)	95 ± 63	101 ± 56	153 ± 132	138 ± 96	58 ± 121	44 ± 88	17.3	0.655	−60.4 to 94.9

*The bolding depicts statistical significance (P < 0.05)*.

**Table 4:** Outcome measures before and after the study and between-group comparisons in the women (mean ± SD).

	**Before**		**After**		Δ		**ANCOVA**		
	**Exp**.	**Con**.	**Exp**.	**Con**.	**Exp**.	**Con**.	**Adj. mean diff**.	***P*-value**	**95% CI**
**STRENGTH**									
Leg press 1-RM (kg)	**88 ± 19**	**95 ± 20**	**99 ± 19**	**96 ± 20**	**12 ± 8**	**1 ± 6**	**5.21**	**0.004**	**1.72 to 8.7**
Leg press peak power (W)	769 ± 221	862 ± 226	893 ± 227	834 ± 219	123 ± 179	−28 ± 90	49.7	0.237	−34 to 133
KE_MVC_ (Nm)	120 ± 27	127 ± 29	130 ± 29	130 ± 33	11 ± 14	3 ± 16	0.9	0.795	−5.9 to 7.8
KE_norm_ (Nm·cm^2^)	4.7 ± 1.4	4.7 ± 0.9	4.9 ± 1.5	4.9 ± 1.2	0.2 ± 0.9	0.2 ± 0.9	−0.1	0.671	−0.6 to 0.4
PF_MVC_ (Nm)	251 ± 49	256 ± 50	263 ± 52	255 ± 51	12 ± 36	−2 ± 26	2.5	0.777	−15.2 to 20.2
PF_norm_ (Nm·cm^2^)	18.0 ± 4.6	17.8 ± 3.1	18.5 ± 4.7	18.3 ± 3.5	0.4 ± 2.4	0.6 ± 2.0	−0.23	0.755	−1.7 to 1.3
**MUSCLE ACTIVITY**									
1-RM VL (μV)	73 ± 41	81 ± 31	85 ± 41	86 ± 25	11 ± 23	6 ± 16	0.02	0.997	−11.2 to 11.2
1-RM VM (μV)	81 ± 50	102 ± 45	96 ± 48	116 ± 30	14 ± 27	14 ± 37	0.06	0.993	−13.6 to 13.7
Power VL (μV)	73 ± 39	79 ± 26	84 ± 39	92 ± 32	11 ± 23	12 ± 17	2.81	0.622	−8.6 to 14.3
Power VM (μV)	85 ± 50	95 ± 42	97 ± 50	115 ± 40	12 ± 39	20 ± 36	−7.91	0.391	−26.3 to 10.5
Voluntary activation (%)	93.3 ± 4.5	95.2 ± 3.0	92.2 ± 5.2	96.0 ± 2.6	−1.1 ± 3.3	0.8 ± 2.4	−0.67	0.550	−2.9 to 1.6
M-wave duration (ms)	11.1 ± 2.5	11.4 ± 3.7	11.0 ± 2.4	10.7 ± 2.6	−0.1 ± 2.5	−0.5 ± 0.9	−0.303	0.641	−1.6 to 1.01
**BODY COMPOSITION**									
Total FFM (kg)	42.3 ± 4.3	40.7 ± 3.7	42.7 ± 4.0	40.7 ± 4.0	0.4 ± 0.8	−2.4 ± 0.7	0.21	0.313	−0.2 to 0.61
Leg FFM (kg)	13.3 ± 1.6	12.9 ± 1.5	13.6 ± 1.6	13.0 ± 1.6	0.3 ± 0.5	0.09 ± 0.4	0.15	0.285	−0.13 to 0.42
Fat mass (kg)	29.2 ± 7.7	24.2 ± 6.4	28.6 ± 7.8	24.0 ± 6.1	−0.6 ± 1.2	−0.2 ± 1.0	−0.04	0.896	−0.66 to 0.58
VL CSA (cm^2^)	**12.8 ± 2.5**	**12.6 ± 2.0**	**13.9 ± 2.7**	**12.6 ± 2.1**	**1.2 ± 1.2**	−**0.02 ± 0.5**	**0.66**	**0.022**	**0.1 to 1.2**
VI CSA (cm^2^)	**15.1 ± 2.7**	**15.1 ± 3.3**	**15.4 ± 2.9**	**14.2 ± 2.6**	−**0.1 ± 3.4**	−**1.0 ± 1.3**	**0.73**	**0.029**	**0.08 to 1.4**
GM CSA (cm^2^)	9.8 ± 2.1	9.8 ± 1.6	10.0 ± 2.1	9.7 ± 1.5	0.2 ± 0.8	−0.1 ± 0.5	0.12	0.560	−0.28 to 0.52
GL CSA (cm^2^)	4.8 ± 1.5	4.7 ± 1.1	4.8 ± 1.4	4.5 ± 0.9	0.01 ± 0.5	−0.2 ± 0.6	0.12	0.340	−0.13 to 0.37
**FUNCTIONAL CAPACITY**									
Forward walk (s)	3.22 ± 0.49	2.95 ± 0.32	3.08 ± 0.42	2.95 ± 0.4	−0.14 ± 0.24	0.01 ± 0.24	0.02	0.735	−0.09 to 0.13
Backward walk (s)	4.93 ± 1.45	4.13 ± 0.9	4.22 ± 1.14	3.86 ± 0.79	−0.71 ± 0.65	−0.27 ± 0.64	0.02	0.885	−0.23 to 0.27
Timed-up-and-go (s)	9.88 ± 1.56	8.94 ± 0.65	9.20 ± 1.26	8.72 ± 0.85	−0.68 ± 0.69	−0.21 ± 0.37	−0.002	0.986	−0.29 to 0.28
Stair climb (s)	3.67 ± 0.86	3.19 ± 0.49	3.45 ± 0.73	3.14 ± 0.51	−0.22 ± 0.24	−0.05 ± 0.16	0.001	0.996	−0.09 to 0.09
Leisure-time PA (min·week)	**105 ± 59**	**115 ± 70**	**121 ± 88**	**263 ± 67**	**14 ± 91**	**123 ± 109**	−**84.7**	**0.005**	−**141 to** −**27**

*The bolding depicts statistical significance (P < 0.05)*.

**Figure 2:**
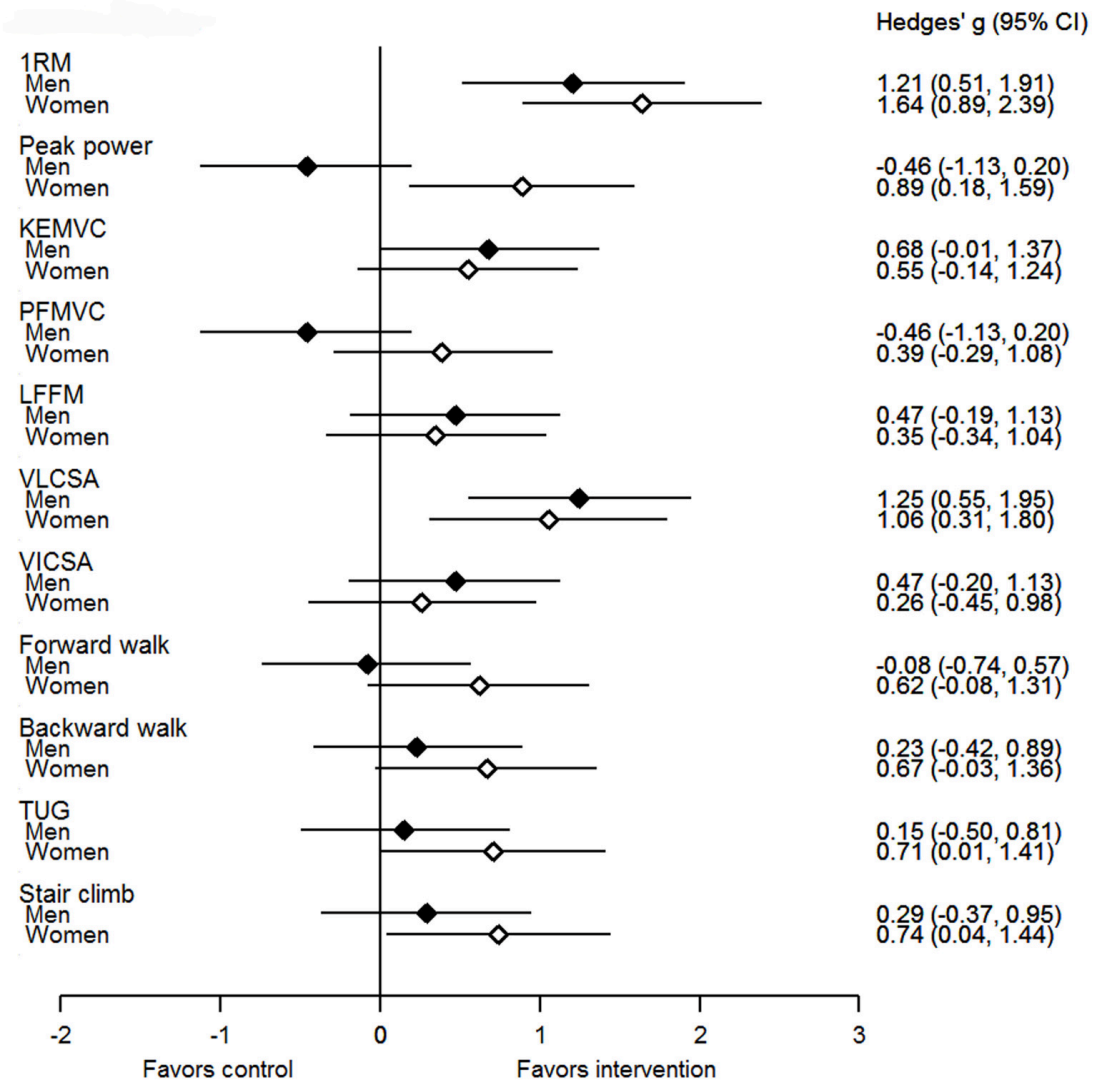
Hedges' *g* effect size (95% confidence intervals) for the unadjusted mean differences between intervention (men and women) and control groups. KEMVC, maximum isometric knee extension torque; PFMVC, maximum isometric plantarflexion torque; LFFM, leg fat-free mass; VLCSA, vastus lateralis cross-sectional area; VICSA, vastus intermedius cross-sectional area; TUG, Timed-Up-and-Go test.

The effect sizes for the mean difference between groups in 1-RM showed large effects favoring intervention in both sexes and a medium effect favoring intervention in KE_MVC_ in men (Figure 2). No significant main effects were observed in isometric plantarflexion torque (absolute or normalized to CSA) or in maximum concentric peak power with 50% 1-RM in men or women (Tables 3, 4). Also, no significant main effects were observed for any surface EMG measurement, voluntary activation level or M-wave duration of the quadriceps in either men or women (Tables 3, 4).

### Functional capacity

As all groups improved functional capacity performance in all tests similarly, no significant main effects were observed in either men or women (Tables 3, 4).

### Body composition and muscle mass

In men (Table 3), a significant main effect for group was observed for leg fat-free mass (*F* = 4.4, *P* = 0.042, 95% CI = 0.02–0.7) with training-induced improvements of 2 ± 5% (*P* = 0.017). A significant main effect for group was also observed in vastus lateralis CSA (*F* = 7.6, *P* = 0.009, 95% CI = 0.2–1.1) with improvements of 6 ± 7% (*P* < 0.001). In women (Table 4), a significant main effect for group was observed for vastus lateralis CSA (*F* = 5.6, *P* = 0.022, 95% CI = 0.1–1.2) with training-induced improvements of 10 ± 10% (*P* < 0.001). A significant main effect for group was also observed for vastus intermedius CSA (*F* = 5.1, *P* = 0.029, 95% CI = 0.1–1.4) with improvements of 3 ± 8% (*P* = 0.040).

The effect sizes for the mean difference between groups in vastus lateralis CSA showed large effects favoring intervention in both sexes and medium and small effects favoring intervention in leg fat-free mass and vastus intermedius CSA in men and women, respectively (Figure 2). There were no significant main effects for total fat-free mass, total fat mass or gastrocnemius medialis and lateralis CSA in men or women (Tables 3, 4).

### Leisure-time physical activity

In women, leisure-time physical activity increased more in the control group compared to the intervention group (mean difference 84.7 min week, *P* = 0.005, 95% CI = 27–141, Table 4). There difference in the change in leisure-time physical activity in the men did not reach the level of statistical significance.

## Discussion

The present study demonstrated that training with moderate-loads and short inter-set rest intervals, aimed to improve muscular endurance, was capable of increasing maximum strength and muscle mass of the knee extensors in previously untrained older men and women. Nevertheless, this type of strength training did not enhance maximum concentric peak power, functional capacity or muscle activation, as measured by surface EMG and the twitch interpolation technique compared to the control group.

Lower-limb maximum strength and muscle mass levels are important to maintain during aging (Raj et al., 2010; Srikanthan and Karlamangla, 2011), and muscle mass is generally accepted to be the greatest predictor of maximum strength (Maughan et al., 1983). It is difficult to compare improvements between studies that utilize different strength tests and training methodologies along with participants that have different ages and physical activity and health backgrounds. Nevertheless, the moderate-load muscular endurance strength training program used in the present study induced statistically significant increases of ~10 and ~14% in 1-RM in men and women, respectively and also ~7% in KE_MVC_ in men. A meta-analysis (Steib et al., 2010) showed that training with heavier loads (>75% 1-RM) elicited greater gains in strength than moderate (55–75% 1-RM) or low (<55% 1-RM) loads. Despite this, other muscular endurance/circuit training interventions have demonstrated improvements of ~22–45% in maximum strength over a 12-week period (Kalapotharakos et al., 2005; Gine-Garriga et al., 2010; Romero-Arenas et al., 2013), which are of a similar magnitude to high-load strength training interventions (12–134%) over 10–12 weeks of training (Häkkinen et al., 1998; Harridge et al., 1999; Walker et al., 2014). An interesting finding was observed by Van Roie et al. (2013), when comparing two different low-load protocols to a high-load protocol. The addition of a higher intensity for the last portion of repetitions led to similar gains in 1-RM as the high-load, low-repetition protocol. One important factor that might separate the present study's findings from those of Van Roie et al. (2013) is the level of neuromuscular fatigue induced by the loading, which is known to influence gains in strength and muscle mass. Lighter loads require a much greater number of repetitions to induce volitional failure, and studies showing similar improvements between high- and low-loads are those with a much greater number of repetitions per set and performed with an unequal amount of total work (e.g., findings of Campos et al., 2002 vs. Mitchell et al., 2012). The present study utilized short inter-set rest intervals rather than a very high number of repetitions per set to progress the level of neuromuscular fatigue taking the participants closer, but not exactly, to volitional failure. Therefore, there was perhaps a lower stimulus for increased strength and muscle mass compared to studies inducing a greater level of neuromuscular fatigue. Ultimately, the results of the present study appear to be at the lower end of the range of improvements in maximum strength.

Regarding muscle hypertrophy, both men and women of the intervention groups in the present study significantly increased their VL CSA by ~6 and ~10%, respectively. Furthermore, leg fat-free mass (~2%) and VI CSA (~3%) significantly increased in men and women, respectively. The VL result is once again a lower magnitude of adaptation than we previously reported (~12–17%, Walker et al., 2014) over 10 weeks of high-load strength training assessed using the same ultrasound methods in older men. It appears that high-loads may be more effective for muscle hypertrophy in previously untrained older individuals (Kalapotharakos et al., 2004; Csapo and Alegre, 2016), however, the CSA gains of the present study are within the range of gains observed from high and moderate-load interventions of previous studies (~10%, Harridge et al., 1999; ~9%, Häkkinen et al., 1998; ~7%, Kalapotharakos et al., 2004). Consequently, statistically significant increases in maximum strength and muscle mass occur during moderate-load muscular endurance strength training in previously untrained older individuals despite being of a slightly lower magnitude compared to high-load strength training. These are positive results of the present study and show that resistance training primarily targeting muscular endurance (along with associated health benefits) can also elicit robust increases in strength and muscle mass, which are the primary advantages of performing strength training over other exercise modes.

Conversely, maximum concentric peak power of the lower limbs assessed by performance with 50% 1-RM load did not demonstrate significant improvements in the present study in either men or women. This finding is in-line with the hypothesis that performing slow, controlled movements during training does not lead to gains in power (Walker et al., 2015). Furthermore, Steib et al. (2010) showed that power training is more effective to improve peak power than controlled velocity strength training, although it should be noted that this result was based on only a few studies. Although the present study found no significant group-level differences in peak power, our muscular endurance training program may have been effective in improving peak power in some individuals. There was a significant negative correlation between baseline peak power and its change during the intervention (*r* = −0.46, *P* < 0.001, *n* = 103). As the older women were weaker than the men at baseline (potentially due to less exposure of strengthening activities in daily living), this may help to explain the favorable effect of intervention observed from Hedge's *g* analyses (*P* < 0.05, Figure 2). These observations raise the possibility that the study may have been underpowered to determine statistically significant between-group differences for this variable (sample size was based on maximum strength and functional capacity estimates) and power development could be a focus of future studies. Nevertheless, since there were no statistically significant differences from control and that power (improvement) is an important determinant of functional capacity (Bassey et al., 1992; Steib et al., 2010), the lack of change in peak power may have influenced our findings regarding functional capacity.

A large number of strength training intervention studies have shown parallel improvements in maximum strength and functional capacity (Schwartz and Evans, 1995; Kalapotharakos et al., 2005; Holviala et al., 2006; Gine-Garriga et al., 2010; Coetsee and Terblanche, 2015; Sundstrup et al., 2016). However, the present study's intervention did not induce a different response compared to control in the four maximum walking speed tests included. It is possible that the performance at baseline in the present study was not a true maximum and that further familiarization sessions may have been needed (Amarante do Nascimento et al., 2013) to extinguish improvements due to learning and confidence. Indeed, Holviala et al. (2006) showed that improvements from a control period of 2 weeks prior to intervention were ~8 and ~5% for maximum 10m walking speed and 10-stair climb tests, respectively. The magnitude of improvement in the intervention groups in the present study (range: −3.8 to −13.2%) were similar compared to results of some previous studies (range −1 to −7%: Holviala et al., 2006; Coetsee and Terblanche, 2015) but lower than others (range −13 to −32%: Kalapotharakos et al., 2005; Gine-Garriga et al., 2010; Sundstrup et al., 2016). Further potential differences between studies that may also partly explain the results could be the age/activity level of the participants, type and duration/number of sessions of strength training, as well as statistical tests used. Another factor that should be considered is the measurement variance and statistical power to detect differences. In the present study, sample size estimates determined that group size should be 22 for an 80% probability level. When separating men and women into different groups, the present study therefore possibly lacked the required number of subjects in the control groups (12 men and 10 women) to detect possible differences.

The present study observed no significant changes in either surface EMG amplitude during leg press 1-RM and maximum concentric power actions or in voluntary activation level assessed by the twitch interpolation technique during isometric knee extension trials. While these methods have been questioned regarding their validity (surface EMG amplitude: Farina et al., 2014) and sensitivity (twitch interpolation technique: Herbert and Gandevia, 1999) to determine adaptation in muscle activation, it is notable that no changes were observed in the present study. Previous studies have observed evidence of increased maximum muscle activation in the knee extensors of older individuals following strength training (Häkkinen et al., 1998; Knight and Kamen, 2001; Walker et al., 2014; Unhjem et al., 2015). However, to the authors' knowledge no other study has investigated the effect of moderate-load muscular endurance strength training on measures of muscle activation. As there were also no changes in M-wave duration, this may indicate that there were no alterations in fiber type composition or propagation of the action potential, which would be considered as muscular adaptation but adaptations within the muscle have been suggested to influence surface EMG amplitude (Arabadziev et al., 2014). Since it is generally accepted that adaptations within the neural system do occur upon the initiation of strength training (Moritani and deVries, 1979), it may be that alterations in muscle activation were specific to the trained action and not necessarily during maximum force or velocity actions. Therefore, further studies are needed in order to fully understand the effects of moderate-load muscular endurance strength training on muscle activation in older adults.

In conclusion, the present study demonstrated that a prolonged period of moderate-load muscular endurance strength training increases maximum strength and muscle mass of the knee extensors in both older men and women. However, this type of strength training was not sufficient to elicit improvements in maximum concentric peak power or muscle activation, as measured by the twitch interpolation technique and surface electromyography or reduce fat mass. Also, this type of strength training did not improve functional capacity as measured by several maximum speed walking tests. Therefore, the present study's muscular endurance strength training intervention did not improve several aspects of age-related health simultaneously.

## Ethics statement

This study was carried out in accordance with the recommendations of the ethical committee of the University of Jyvaskyla, Finland with written informed consent from all subjects. All subjects gave written informed consent in accordance with the Declaration of Helsinki. The protocol was approved by the ethical committee of the University of Jyvaskyla, Finland.

## Author contributions

Conceived and designed the experiments: SW, GH, KH, RN. Performed experiments and analyzed data: SW. Interpreted results of research: SW, GH, KH, RN. Drafted, edited, critically revised paper and approved final version of manuscript: SW, GH, KH, RN.

### Conflict of interest statement

The authors declare that the research was conducted in the absence of any commercial or financial relationships that could be construed as a potential conflict of interest.

## References

[B1] Amarante do Nascimento M. Januario R. S. Gerage A. M. Mayhew J. L. Cheche Pina F. L. Cyrino E. S. (2013). Familiarization and reliability of one repetition maximum strength testing in older women. J. Strength Cond. Res. 27, 1636–1642. 10.1519/JSC.0b013e3182717318 22990569

[B2] Arabadziev T. I. Dimitrov V. G. Dimitrov G. V. (2014). The increases in surface EMG could be a misleading measure of neural adaptation during the early gains in strength. Eur. J. Appl. Physiol. 114, 1645–1655. 10.1007/s00421-014-2893-y 24789744

[B3] Bassey E. J. Fiatarone M. A. O'Neill E. F. Kelley M. Evans W. J. Lipsitz L. A. (1992). Leg extensor power and functional performance in very old men and women. Clin. Sci. 82, 321–327. 1312417 10.1042/cs0820321

[B4] Campos G. E. Luecke T. J. Wendeln H. K. Toma K. Hagerman F. C. Murray T. F.. (2002). Muscular adaptations in response to three different resistance-training regimens: specificity of repetition maximum training zones. Eur. J. Appl. Physiol. 88, 50–60. 10.1007/s00421-002-0681-6 12436270

[B5] Clayton N. Drake J. Larkin S. Linkul R. Martino M. Nutting M. (2015). Foundations of Fitness Programming. Colorado Springs, CO: National Strength and Conditioning Association.

[B6] Coetsee C. Terblanche E. (2015). The time course of changes induced by resistance training and detraining on muscular and physical function in older adults. Eur. Rev. Aging Phys. Act. 12:7. 10.1186/s11556-015-0153-8 26865871 PMC4748325

[B7] Csapo R. Alegre L. M. (2016). Effects of resistance training with moderate vs heavy loads on muscle mass and strength in the elderly: a meta-analysis. Scand. J. Med. Sci. Sports 26, 995–1006. 10.1111/sms.12536 26302881

[B8] Farina D. Merletti R. Enoka R. M. (2014). The extraction of neural strategies from the surface EMG: an update. J. Appl. Physiol. 117, 1215–1230. 10.1152/japplphysiol.01070.2003 25277737 PMC4254845

[B9] Fiatarone M. A. Marks E. C. Ryan N. D. Meredith C. N. Lipsitz L. A. Evans W. J. (1990). High-intensity strength training in nonagenarians. effects on skeletal muscle. J. Am. Med. Assoc. 263, 3029–2034. 2342214

[B10] Gine-Garriga M. Guerra M. Pages E. Manini T. M. Jimenez R. Unnithan V. B. (2010). The effect of functional circuit training on physical frailty in frail older adults: a randomized controlled trial. J. Aging Phys. Act. 18, 401–424. 10.1123/japa.18.4.401 20956842

[B11] Häkkinen K. Newton R. U. Gordon S. E. McCormick M. Volek J. S. Nindl B. C.. (1998). Changes in muscle morphology, electromyographic activity, and force production characteristics during progressive strength training in young and older men. J. Gerontol A Biol. Sci. Med. Sci. 53, B415–B423. 9823737 10.1093/gerona/53a.6.b415

[B12] Harridge S. D. Kryger A. Stensgaard A. (1999). Knee extensor strength, activation, and size in very elderly people following strength training. Muscle Nerve 22, 831–839. 10398199 10.1002/(sici)1097-4598(199907)22:7<831::aid-mus4>3.0.co;2-3

[B13] Henwood T. R. Taaffe D. R. (2005). Improved physical performance in older adults undertaking a short-term programme of high-velocity resistance training. Gerontology 51, 108–115. 10.1159/000082195 15711077

[B14] Herbert R. D. Gandevia S. C. (1999). Twitch interpolation in human muscles: mechanisms and implications for measurement of voluntary activation. J. Neurophysiol. 82, 2271–2283. 10561405 10.1152/jn.1999.82.5.2271

[B15] Holviala J. H. Sallinen J. M. Kraemer W. J. Alen M. Häkkinen K. (2006). Effects of strength training on muscle strength characteristics, functional capabilities, and balance in middle-aged and older women. J. Strength Cond. Res. 20, 336–344. 10.1519/R-17885.1 16686561

[B16] Kalapotharakos V. I. Michalopoulos M. Godolias G. Tokmakidis S. P. Malliou P. V. Gourgoulis V. (2004). The effects of high- and moderate-resistance training on muscle function in the elderly. J. Aging Phys. Act. 12, 131–143. 10.1123/japa.12.2.131 15223882

[B17] Kalapotharakos V. I. Michalopoulos M. Tokmakidis S. P. Godolias G. Gourgoulis V. (2005). Effects of a heavy and a moderate resistance training on functional performance in older adults. J. Strength Cond. Res. 19, 652–657. 10.1519/15284.1 16095421

[B18] Knight C. A. Kamen G. (2001). Adaptations in muscular activation of the knee extensor muscles with strength training in young and older adults. J. Electromyogr. Kinesiol. 11, 405–412. 10.1016/S1050-6411(01)00023-2 11738953

[B19] Liu C. J. Latham N. K. (2009). Progressive resistance strength training for improving physical function in older adults. Cochrane Database Syst. Rev. 3:CD002759 10.1002/14651858.CD002759 PMC4324332 19588334

[B20] Lovell D. I. Cuneo R. Gass G. C. (2010). The effect of strength training and short-term detraining on maximum force and the rate of force development of older men. Eur. J. Appl. Physiol. 109, 429–435. 10.1007/s00421-010-1375-0 20140683

[B21] Maughan R. J. Watson J. S. Weir J. (1983). Strength and cross-sectional area of human skeletal muscle. J. Physiol. 338, 37–49. 6875963 10.1113/jphysiol.1983.sp014658 PMC1197179

[B22] Merton P. A. (1954). Voluntary strength and fatigue. J. Physiol. 123, 553–564. 13152698 10.1113/jphysiol.1954.sp005070 PMC1366225

[B23] Mitchell C. J. Churchward-Venne T. A. West D. W. Burd N. A. Breen L. Baker S. K. (2012). Resistance exercise load does not determine training-mediated hypertrophic gains in young men. J. Appl. Physiol. 113, 71–77. 10.1152/japplphysiol.00307.2012 22518835 PMC3404827

[B24] Moritani T. deVries H. A. (1979). Neural factors versus hypertrophy in the time course of muscle strength gain. Am. J. Phys. Med. 58, 115–130. 453338

[B25] Raj I. S. Bird S. R. Shield A. J. (2010). Aging and the force-velocity relationship of muscles. Exp. Gerontol. 45, 81–90. 10.1016/j.exger.2009.10.013 19883746

[B26] Romero-Arenas S. Blazevich A. J. Martínez-Pascual M. Pérez-Gómez J. Luque A. J. López-Román F. J.. (2013). Effects of high-resistance circuit training in an elderly population. Exp. Gerontol. 48, 334–340. 10.1016/j.exger.2013.01.007 23352954

[B27] Rosenberg J. G. Ryan E. D. Sobolewski E. J. Scharville M. J. Thompson B. J. King G. E. (2014). Reliability of panoramic ultrasound imaging to simultaneously examine muscle size and quality of the medial gastrocnemius. Muscle Nerve 49, 736–740. 10.1002/mus.24061 24038069

[B28] Schwartz R. S. Evans W. J. (1995). Effects of exercise on body composition and functional capacity of the elderly. J. Gerontol. A Biol. Sci. Med. Sci. 50, 147–150. 7493209 10.1093/gerona/50a.special_issue.147

[B29] Srikanthan P. Karlamangla A. S. (2011). Relative muscle mass is inversely associated with insulin resistance and prediabetes. findings from the third National Health and Nutrition Examination Survey. J. Clin. Endocrinol. Metabol. 96, 2898–2903. 10.1210/jc.2011-0435 21778224

[B30] Steib S. Schoene D. Pfeifer K. (2010). Does-response relationship of resistance training in older adults: a meta-analysis. Med. Sci. Sports Exerc. 42, 902–914. 10.1007/s40279-015-0385-9 19996996

[B31] Strasser B. Siebert U. Schobersberger W. (2010). Resistance training in the treatment of the metabolic syndrome: a systematic review and meta-analysis of the effect of resistance training on metabolic clustering in patients with abnormal glucose metabolism. Sports Med. 40, 397–415. 10.2165/11531380-000000000-00000 20433212

[B32] Sundstrup E. Jakobsen M. D. Andersen L. L. Andersen T. R. Randers M. B. Helge J. W.. (2016). Positive effects of 1-year football and strength training on mechanical muscle function and functional capacity in elderly men. Eur. J. Appl. Physiol. 116, 1127–1138. 10.1007/s00421-016-3368-0 27068158

[B33] Taaffe D. R. Pruitt L. Pyka G. Guido D. Marcus R. (1996). Comparative effects of high- and low-intensity resistance training on thigh muscle strength, fiber area, and tissue composition in elderly women. Clin. Physiol. 16, 381–392. 8842574 10.1111/j.1475-097x.1996.tb00727.x

[B34] Tanimoto M. Ishii N. (2006). Effects of low-intensity resistance exercise with slow movement and tonic force generation on muscular function in young men. J. Appl. Physiol. 100, 1150–1157. 10.1152/japplphysiol.00741.2005 16339347

[B35] Unhjem R. Lundestad R. Fimland M. S. Mosti M. P. Wang W. (2015). Strength training-induced responses in older adults: attenuation of descending neural drive with age. AGE 37:9784. 10.1007/s11357-015-9784-y 25940749 PMC4418975

[B36] Van Roie E. Delecluse C. Coudyzer W. Boonen S. Bautmans I. (2013). Strength training at high versus low external resistance in older adults: effects on muscle volume, muscle strength, and force-velocity characteristics. Exp. Gerontol. 48, 1351–1361. 10.1016/j.exger.2013.08.010 23999311

[B37] Walker S. Blazevich A. J. Haff G. G. Tufano J. J. Newton R. U. Häkkinen K. (2016). Greater strength gains after training with accentuated eccentric than traditional isoinertial loads in already strength-trained men. Front. Physiol. 27:149 10.3389/fphys.2016.00149 PMC4847223 27199764

[B38] Walker S. Peltonen H. Häkkinen K. (2015). Medium-intensity, high-volume “hypertrophic” resistance training did not induce improvements in rapid force production in healthy older men. AGE 37:9786 10.1007/s11357-015-9786-9 25911469 PMC4409589

[B39] Walker S. Peltonen H. Sautel J. Scaramella C. Kraemer W. J. Avela J.. (2014). Neuromuscular adaptations to constant vs. variable resistance training in older men. Int. J. Sports Med. 35, 69–74. 10.1055/s-0033-1343404 23825004

